# Development of a Digital RT-PCR Method for Absolute Quantification of Bluetongue Virus in Field Samples

**DOI:** 10.3389/fvets.2020.00170

**Published:** 2020-04-21

**Authors:** Angela M. Rocchigiani, Maria G. Tilocca, Ottavio Portanti, Bruna Vodret, Roberto Bechere, Marco Di Domenico, Giovanni Savini, Alessio Lorusso, Giantonella Puggioni

**Affiliations:** ^1^Department of Sanità Animale, Istituto Zooprofilattico Sperimentale Della Sardegna, Sassari, Italy; ^2^OIE Reference Laboratory for Bluetongue, Istituto Zooprofilattico Sperimentale Abruzzo e Molise, Teramo, Italy

**Keywords:** Bluetongue, Reoviridae, RNA quantification, droplet digital RT-PCR, Quantitative Real Time -RT- PCR

## Abstract

Bluetongue (BT) is a major Office International des Epizooties (OIE)-listed disease of wild and domestic ruminants caused by several serotypes of Bluetongue virus (BTV), a virus with a segmented dsRNA genome belonging to the family *Reoviridae*, genus *Orbivirus*. BTV is transmitted through the bites of *Culicoides* midges. The aim of this study was to develop a new method for quantification of BTV Seg-10 by droplet digital RT-PCR (RTdd-PCR), using nucleic acids purified from complex matrices such as blood, tissues, and midges, that notoriously contain strong PCR inhibitors. First, RTdd-PCR was optimized by using RNAs purified from serially 10-fold dilutions of a BTV-1 isolate (10^5.43^TCID_50_/ml up to 10^−0.57^ TCID_50_/ml) and from the same dilutions spiked into fresh ovine EDTA-blood and spleen homogenate. The method showed a good degree of linearity (*R*^2^ ≥ 0.995). The limit of detection (LoD) and the limit of quantification (LoQ) established were 10^−0.67^TCID_50_/ml (0.72 copies/μl) and 10^0.03^TCID_50_/ml (3.05 copies/μl) of BTV-1, respectively. Second, the newly developed test was compared, using the same set of biological samples, to the quantitative RT-PCR (RT-qPCR) detecting Seg-10 assay widely used for the molecular diagnosis of BTV from field samples. Results showed a difference mean of 0.30 log between the two assays with these samples (*p* < 0.05). Anyway, the analysis of correlation demonstrated that both assays provided similar measurements with a very close agreement between the systems.

## Introduction

Digital polymerase chain reaction (dPCR) is a recent technology enables an accurate absolute quantification of target nucleic acids. The principle of dPCR was first described in the 1990s (1, 2). The dPCR approach combines limiting dilutions, PCR, and Poisson distribution to quantitate the total number of amplifiable targets within a sample (1).

Digital technology is based on end-point PCR that provides the direct measure of nucleic acids without relying on a standard curve (3). In a dPCR assay, the sample is randomly partitioned into individual reactions, such that some contain no nucleic acid template and others contain one or more template copies (4). The very high number of partitions of the sample allows a significant precision on results (5).

After end-point PCR amplification, each partition is analyzed and distinct as positive (presence of PCR products) or negative (absence of PCR products). The fraction of amplification positive partitions is used to estimate the concentration of the initial target sequence using binomial Poisson statistics (4, 6). Nowadays, different dPCR commercial platforms are available as a useful tool for precise quantification of nucleic acids in a variety of basic research and clinical applications (4, 7–9).

Despite the fact that quantitative PCR or real-time PCR (qPCR) has been widely utilized to quantify nucleic acid in many areas of research and diagnostics tests, it has same disadvantages such as the necessity for a standard curve, the lack of universal standards of known quantity, and also the efficiency can be influenced by many factors including inhibitors. The qPCR is the gold standard for molecular quantitation in viral diagnostics; dPCR offers several potential advantages over qPCR (10). Digital PCR uses an amplification reaction system similar to a system of standard qPCR, but does not require the same level of calibration or controls as traditionally used in qPCR (5).

Digital PCR overcomes the need for a standard curve, and it is increasingly used for DNA/RNA viral quantification, in human and animal health (11–17). Bluetongue (BT) is an Office International des Epizooties (OIE)-listed infectious disease of domestic and wild ruminants (18), transmitted mainly through the bites of *Culicoides* midges. Bluetongue virus (BTV), which belongs to the genus Orbivirus of the family Reoviridae, causes significant economic losses due to mortality, decline in production, and restrictions on trade in animals from infected areas (19). BTV genome consists of 10 linear double strand segments (Seg-1 to Seg-10), encoding seven structural proteins (VP1-Vp7) and five non-structural proteins (NS1 to NS4 and S10-ORF2) involved in viral replication, morphogenesis, and assembly processes (20).

Currently, there are 24 classic serotypes of the virus, all capable of causing BT, plus a series of new serotypes, defined as atypical because infected animals are asymptomatic (21–27).

For molecular diagnostic laboratories, OIE recommends the use of a real-time RT-PCR (RT-PCR_NS3_) assay in order to confirm clinical cases, to establish uninfected animals before handling, to check the prevalence of infection, and for surveillance purposes.

The method real-time RT-PCR_NS3_ (21) allows to detect all circulating known BTV serotypes, by retro-transcription and amplification of a region of segment 10 of the viral RNA, coding for a non-structural NS3 protein, purified by blood-EDTA, biological liquid, and organ tissues taken from susceptible species and by hematophagous insects. RT-PCR targeting in Seg-2 coding for a least conserved virion outer capsid protein (VP2) identifies the specific BTV serotype (28, 29).

Because of its accuracy and precision, real-time quantitative RT-qPCR is the method of choice when quantitative analysis is required. However, there are no available reference certificated material (standard) for the quantification of bluetongue virus. Toussaint et al. (30) used recombinant plasmid obtained by inserting BTV PCR product into PCRII-TOPO vector by TA-cloning and RNA synthesized *in vitro* with Riboprobe system T7 (Promega). (31) employed RiboMax Large scale RNA production System (Promega) for transcription of standard RNA by bluetongue recombinant plasmid PGEM –T Easy Vector RNA. Maan et al. (32) transcribed from recombinant pGEMT plasmid RNA BT using the mMessage mMachine T7 Ultra Kit (Life Technologies). Overall, the use of different calibration standards in different performing assays can lead to non-reproducible results between laboratories, even when testing the same material (12, 33).

To overcome these limitations, we aimed to develop a new method for quantification of BTV Seg-10 by droplet digital RT-PCR (RT-dd-PCR), using nucleic acids purified from complex matrices such as blood, tissues, and midges, that notoriously contain strong PCR inhibitors. The RT-PCR_NS3_ method recommended by OIE was transferred to the digital platform, optimized by using RNAs purified from serially 10-fold dilutions of a BTV-1 isolate, spiked into fresh ovine EDTA-blood and spleen homogenate. The limit of detection (LoD) and the limit of quantification (LoQ) were established by using serial dilutions of BTV-1 RNA purified. Using RNAs purified from field samples, the newly developed assay was compared, with the RT-qPCR _NS3_ detecting the same target Seg-10.

## Materials and Methods

### Virus Strain, Spiked-In Samples, and Field Samples Collection

BTV-1/2006 strain, isolated from the spleen of an infected sheep that succumbed during the BTV-1 outbreak in Sardinia (Italy) during 2006, was employed for the study. The BTV-1/2006 strain was titrated by end-point onto VERO cells by Sperman/Karber method (10^5.43^/TCID_50_/ml). Four 10-fold serial dilutions of BTV-1 suspensions (from 10^2.43^ to 10^−0.57^ TCID_50_/ml) spiked into ovine blood samples and in ovine spleen homogenates (10% w/v) were used to evaluate possible inhibition caused by matrices. The optimized droplet digital RT-PCR (RT-ddPCR) was finally evaluated on a total of 44 field samples tested positive for real-time RT-PCR_NS3_, including 16 of *Culicoides imicola* and 28 of ovine blood EDTA. *Culicoides* samples were collected in farms of southern Sardinia, during entomological surveillance, by the national surveillance plan, in the years 2017 and 2018. The blood samples were collected from farms located in the same part of the region. Four negative blood samples were used as negative controls. The blood samples were refrigerated at 5° ± 3°C. *C. imicola* samples were stored at −70 ± 10°C until the time of processing and analysis. The results were compared with those obtained by the RT-qPCR_NS3_.

### Nucleic Acid Purification

RNA purification from viral suspensions, spiked-in samples, and field samples, mosquitos included, was performed by MagMax Core Nucleic Acid Purification Kit (Applied Biosystems-ThermoFisher Scientific-USA) in automated sample preparation workstation MagMAX Express 96 (Applied Biosystems) according to the manufacturer's instructions. Primers and probe were the same published by OIEb (34), which amplify a portion of Seg-10. This RT-PCR_NS3_ assay is widely used for molecular diagnosis of BTV. In order to optimize RT-ddPCR assay, the same set of primers and probe were used in all experiments.

### Droplet Digital RT-PCR (RT-ddPCR) Optimization

Purified nucleic acids were quantified using QX200™ Droplet Digital PCR System (BioRad Laboratories, USA). The assay was performed in 20 μl using the One-step RT-ddPCR Advanced Kit for Probe (Bio-Rad) consisting of: 5 μl supermix 4 X, 2 μl of reverse transcriptase (20 U/μl), 1 μl of 300 mM DTT, 2 μl of RNA template, and the primers and probe at final concentrations of 0.9 and 0.25 μM, respectively, according to the manufacturer's instructions. In order to optimize RT-ddPCR assay, primers and probe were further tested at different concentrations within the range of 0.4–0.9 μM and 0.15–0.25 μM by using RNA purified from different BTV-1 suspensions (titer 10^2.43^, 10^1.43^, 10^0.43^, 10^−0.57^ TCID_50_/ml). RNA was denatured with primers for 5 min at 95°C, stabilized for 3 min at 4°C, and then added to the RT-ddPCR mixture reaction as indicated above. No template controls (NTC) were used for monitoring primer-dimer formation and contaminations. Twenty microliters RT-ddPCR mixture/sample were placed in each well of droplet generator DG8 cartridge (BioRad Laboratories, USA) with 70 μl of droplet generator oil (BioRad Laboratories, USA) and emulsified in QX-200 Droplet Generator (BioRad Laboratories, USA) partitioning in 20,000 water in oil nanoliter-size droplet. Then, a volume of 40 μl of emulsion/sample was transferred to a 96-well reaction plate (Eppendorf, Hauppauge, NY), heat-sealed with pierceable foil sheets by the PX1™ PCR Plate Sealer (BioRad Laboratories, USA), and amplified in C1000 Touch™ Thermal Cycler (BioRad Laboratories, USA). So as to allow an optimal distinction between positive and negative droplets, PCR annealing temperature was optimized by thermal gradient from 55 to 65°C. Cycling conditions were the following: 48°C for 30 min, 95°C for 2 min, followed by 50 cycles of 95°C for 15 s, 55C-65°C for 30 s, and 72°C for 30 s. At the end of amplification, the PCR plates were read by the QuantaSoft Droplet Reader (BioRad Laboratories, USA) that measures the fluorescence intensity of each droplet and detects the size and shape as droplets pass detector. The absolute concentration of each sample was automatically reported as copy number of Seg-10 of BTV/μl by the ddPCR QuantaSoft Software V.1.7.4.0917 (BioRad Laboratories, USA) by calculating the ratio of the positive droplets over the total droplets combined with Poisson distribution with 95% confidence interval.

### Performance of RT-ddPCR Assay

Linearity of the assay was defined by using 10-fold dilutions of BTV1-RNA purified (ranging from 10^2, 43^ TCID_50_/ml up to 10^−0, 57^ TCID_50_/ml), analyzed in seven replicates, with optimized conditions of primers, probe, and amplification program. The range of linearity was defined by plotting the log value of titers (TCID_50_/ml) BTV-1 dilution against the absolute measured value (copies/μl). The LoD and LoQ were evaluated by using 20 replicates of 5-fold serial dilutions of BTV-1 RNA purified (ranging from 10^1.43^ to 10^−1.37^ TCID_50_/ml). The LoD of RT-ddPCR was determined as the last serial dilution detected in 95% of replicates, whereas the LoQ was set at the lowest dilution showing a coefficient of variation percentage below the threshold (CV% = 25) for acceptance criteria of quantitative methods (35, 36). Furthermore, to evaluate the intra-assay and inter-assay repeatability, three different dilutions of BTV-1 (10^2.43^, 10^1.43^, 10^0.43^ TCID_50_/ml) were tested in seven replicates in two different days; the CV% was then considered. pGEM T-easy vector (Promega, Milan-Italy) carrying Seg-10 of a BTV-1 strain in serial dilutions, from 2 × 10^4^ to 2 × 10^1^ copies, was also used to evaluate accuracy of the RT ddPCR for quantification purposes. Finally, matrix effect was evaluated comparing *R*^2^ values of RNA isolated from four BTV-1 dilutions (from 10^2.43^ to 10^−0.57^ TCID_50_/ml), and RNA isolated from four blood and four spleen homogenates spiked (10% w/v) with the same BTV-1 TCID_50_/ml.

### Quantitative Real Time Assay (RT-qPCR_NS3_)

RT-qPCR_NS3_ assay, as described above, was performed using the 7900HT Fast Real-Time PCR System (Applied Biosystems) and SuperScript™ III Platinum™ One-Step qRT-PCR Kit (ThermoFisher- Life Technologies). RNA was denatured with 0.4 μM primers for 5 min at 95°C, stabilized for 3 min at 4°C, and then added to the RT-qPCR mixture reaction as indicated above. The one step RT-qPCR mixture was prepared in 25 μl reaction volume, 12.5 μl 2X Reaction Mix, 0.5 μl of 50X ROX Reference Dye, 1 μl Mg_2_SO_4_ 50 mM, 0.5 μl SSIII RT-Platinum™ Taq Mix, 0.2 μM probe, and 2 μl of RNA. Cycling conditions were the following: 48°C for 30 min, 95°C for 2 min, followed by 50 cycles of 95°C for 15 s, 60°C for 30 s, and 72°C for 30 s. In order to assess the standard curve the pGEM T-easy vector (Promega, Milan-Italy) carrying Seg-10 of a BTV-1 strain was employed, available at IZSAM (pGEM-BTV-NS3, 10^8^ copies/μl). The standard curve was constructed placing Cq values of seven serial 10-fold dilutions of pGEM-BTV-NS3 (1 × 10^7^ copies/μl up to 1 × 10^1^ copies/μl), performed in triplicate wells, against the log value of the number of copies of BTV-1 Seg-10. BTV-1 Seg-10 copy number in each sample was determined by Cq value to the standard curve. Cq value was generated by 7900 Software SDS 2.4.1 (Applied Biosystems). Amplification Efficiency and *R*^2^ of the calibration curve were calculated.

### Comparison of RT-ddPCR and RT-qPCRNS3 Assays for Quantitation of BTV-1 Seg-10 in Field Samples

To evaluate the performance of RT-ddPCR against the RT-qPCR_NS3_, 44 field samples were tested in triplicate wells with both assays and the difference of log of quantification was evaluated to verify the agreement between two assays.

### Statistical Analysis

Data were converted into a logarithmic format. Linear regression analyses were conducted using Microsoft Excel 2010. In order to compare quantification by RT-ddPCR and RT-qPCR_NS3_ statistical analysis was conducted by Statgraphics 18 Centurion Software (Version 18.1.06).

## Results

### Optimization of RT-ddPCR

**Figure 1:**
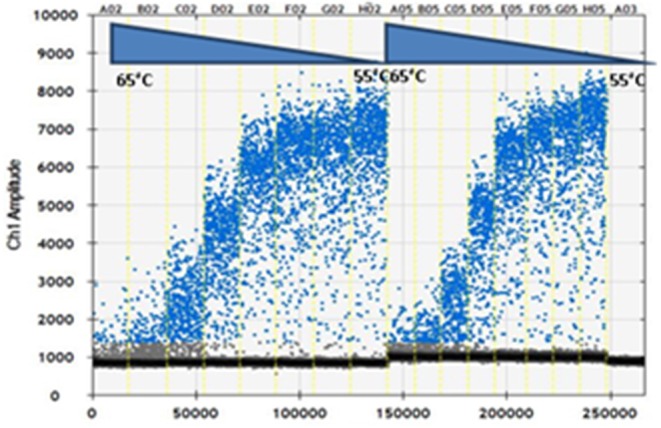
Optimization of droplet digital RT-PCR (RT-ddPCR). Fluorescence amplitude plotted against annealing temperature gradient for 0.7 μM primers (lanes A02-H02) and 0.9 μM primers (lanes A05-H05): 65, 64.3, 63, 61.3, 58.8, 56.9, 55.7, and 55°C; 0.25 μM probe. Lane A03 No Template Control (NTC).

The primers and probe concentrations were optimized by using RNA purified from dilution of BTV-1 from 10^2.43^ to 10^−0.57^. As shown in Figure 1, the optimal primers and probe concentrations were 0.9 and 0.25 μM per reaction, respectively, i.e., the highest concentrations, among those tested, as recommended by the manufacturer (Biorad). PCR annealing temperature was optimized by thermal gradient from 55 to 65°C. The optimum annealing temperature was at 58.8°C, which resulted in the greatest difference of fluorescence amplitude between positive and negative droplets. The optimal cycling conditions were: 48°C for 30 min, 95°C for 2 min, followed by 50 cycles of 95°C for 15 s, 58.8°C for 30 s, and 72°C for 30 s, 1 cycle of 98°C for 10 min essential for droplet stabilization and infinite 12°C hold. It was used a 2.5°C/s ramp rate to ensure each droplet reached the correct temperature for each step during the cycling. The parameters above were used in following RT-ddPCR experiments of our study.

### Performance of RT-ddPCR Assay

**Figure 2:**
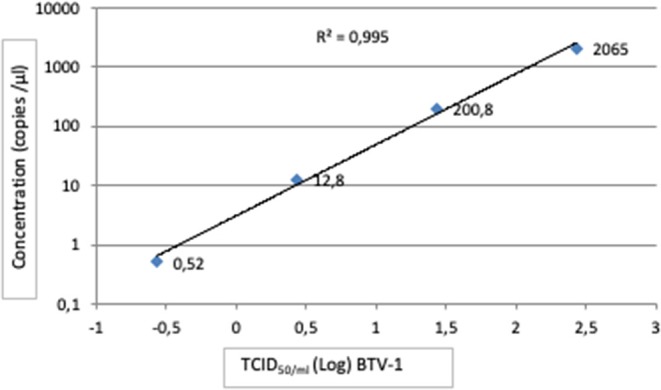
Linear regression of RT-ddPCR assay using RNA extracted from 10-fold dilution of BTV-1/2006 from TCID_50_ 10^2.43^ to 10^−0.57^, analyzed in seven replicates, at the final optimized conditions.

**Figure 3:**
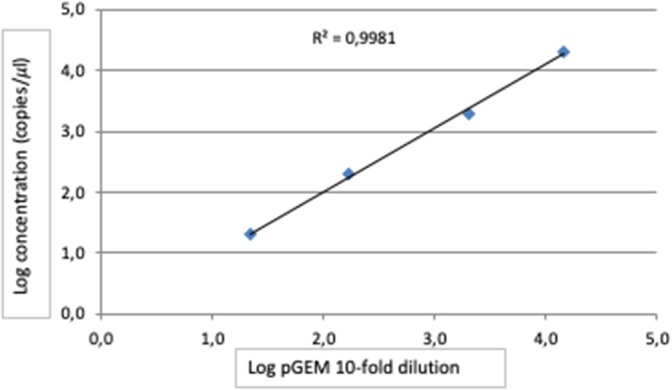
Linear regression of RT-ddPCR assay using four 10-fold dilution of pGEM-BTV-NS3 from 2 × 10^4^ to 2 × 10^1^, analyzed in three replicates, at the final optimized conditions.

The trend line of detection BTV-1 concentration by RT-ddPCR exhibited a good degree of linearity (*R*^2^ ≥ 0.995) in the range from 10^2.43^ TCID_50_/ml to 10^−0.57^ TCID_50_/ml (Figure 2). According to the definition, the LoD established was 10^−0.67^ TCID_50_/ml BTV1 corresponding to 0.72 copies/μl (Table 1). Conversely, the LoQ determined was 10^0.03^ TCID_50_/ml of BTV-1 (3.05 copies/μl) with a CV% value 21 (Table 1). Results of the copy number obtained by RT-ddPCR relative to the four pGEM 10-fold dilutions give a good degree of linearity (*R*^2^ = 0.998), especially in the range 20–2000 copies as reported in Table 2 and Figure 3. The CV% values were also considered to assess repeatability. The analysis of the seven replicates for the three dilutions gave back CV% lower than the threshold (CV% = 25) in all cases for both intra- and inter-assay (Table 3).

**Table 1:** Limit of detection (LoD) and limit of quantification (LoQ) of droplet digital RT-PCR (RT-ddPCR).

**BTV-1 Log_**10**_ TCID_**50/ml**_**	**Mean values (copies/μl) ± SD^a^**	**CV (%)^b^**
1.43	195 ± 4.35	2.23
0.73	29 ± 1.17	4.01
0.03	3.05 ± 0.64^c^	21
−0.67	0.72 ± 0.40^d^	25
−1.37	0.09 ± 0.09	91

a *Mean values of copies number BTV-1 in μl detected by RT-ddPCR and standard deviation*.

b *Coefficient of variation*.

c *LoQ*.

d *LoD*.

**Table 2:** pGEM detection of the RT-ddPCR.

**Concentration of pGEM (copies/2 μl)**	**Concentration of pGEM (log)**	**Detected concentration copies**	**Detected concentration (log)**
20000	4.30	14220	4.15
2000	3.30	1997	3.30
200	2.30	169	2.23
20	1.30	22	1.34

**Table 3:** Repeatability of RT-ddPCR assay.

	**Intra-assay**		**Inter-assay**	
**Concentration of BTV-1 (TCID _**50**_/ml)**	**Mean values of seven replicates (copies /μl) ± SD^a^**	**CV (%)^b^**	**Mean values of seven replicates (copies /μl) ± SD^a^**	**CV (%)^b^**
1 × 10 ^2.43^	2066.43 ± 53.05	2.56	2017.43 ± 41.35	2.05
1 × 10 ^1.43^	200.86 ± 5.61	2.79	200.71 ± 8.47	4.22
1 × 10 ^0.43^	12.84 ± 1.62	12.62	12.07 ± 1.26	10.51

a *Mean values of copies number BTV-1 in μl of seven replicates detected by RT-ddPCR and standard deviation*.

b *Coefficient of variation*.

### Evaluation Matrix-Effect of RT-ddPCR

**Figure 4:**
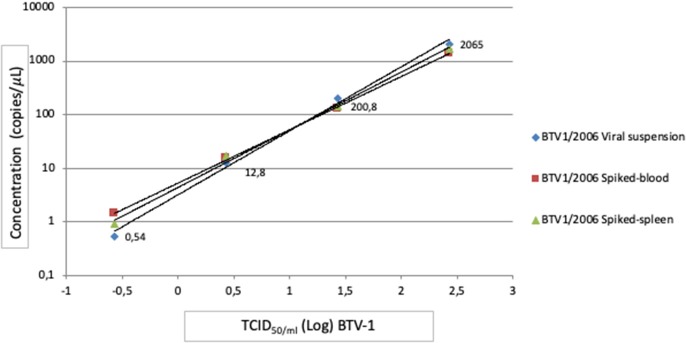
RT-ddPCR Matrix effect. Linear regression analysis of 10-fold serial dilutions of BTV-1/2006, blood and spleen spiked with the same BTV-1/2006 TCID_50_/ml. Blue rhombus BTV-1/2006 viral suspension *R*^2^ = 0.9954; Green triangle BTV-1/2006 spiked-spleen *R*^2^ = 0.9964; Red square BTV-1/2006 spiked-blood *R*^2^ = 0.9996.

BTV-1 suspensions from 10^2.43^ to 10^−0.57^ TCID_50_/ml spiked in blood and in spleen tissue showed good resilience to inhibitor blood and tissue factors compared to same viral suspensions without matrix, in linear range of the RT-ddPCR assay. As indicated in Figure 4 quantitative linearity analysis in matrices showed a good linearity with *R*^2^ close to 1.

### Comparison of RTdd-PCR and RT-qPCRNS3 Assays for Quantitation of Seg-10 of BTV in Field Samples

**Figure 5:**
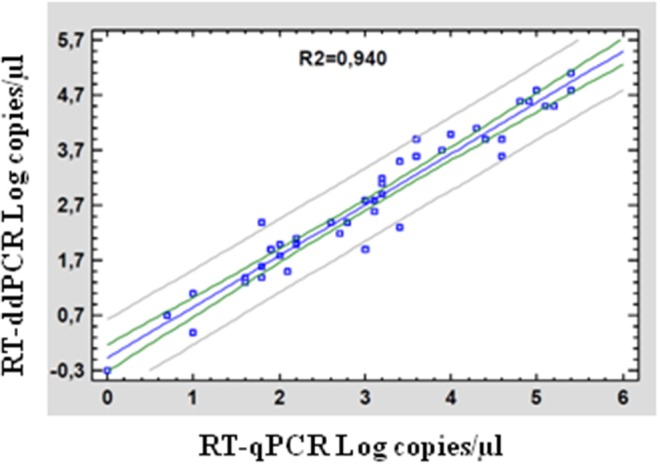
Correlations between real-time RT-qPCR_NS3_ and ddPCR for field samples. Fit regression model (blue). 95% Confidence limits (gray). 95% Prediction limits (green).

The two assays were compared calculating the difference between the logarithm of RT-qPCR_NS3_ quantification, i.e., copies of Seg-10 of BTV /μl of sample and the RT-ddPCR correspondent data. In field samples, the log difference average was 0.30 (*p* = 0.04968), in detail of 0.20 and 0.40, respectively, for blood EDTA samples and *Culicoides* midge as shown in Table 4 and Table S1. Figure 5 shows the correlations between the log copies of Seg-10 of BTV/μl of sample in RT-ddPCR with those in RT-qPCR_NS3._

**Table 4:** Comparison of RT-qPCRNS3 and RT-ddPCR assays for quantitative detection of BTV in field samples.

	**Real time RT-qPCRNS_**3**_**		**RT-ddPCR**		
**Field sample^a^**	**Copies BTV/μl sample^b^**	**Log (copies/μl)^c^**	**Copies BTV/μl sample^d^**	**Log (copies/μl)^e^**	**Log difference^f^**
Culicoides 79633/1	9701.38	4.0	9787.50	4.0	0.0
Culicoides 79633/3	7277.90	3.9	5400.00	3.7	0.2
Culicoides 79633/5	91433.73	5.0	60356.25	4.8	0.2
Culicoides 79633/8	75950.56	4.9	38812.00	4.6	0.3
Culicoides 79633/9	242211.14	5.4	116325.00	5.1	0.3
Culicoides 79633/10	1226.22	3.1	387.00	2.6	0.5
Culicoides 79633/11	70.02	1.8	38.50	1.6	0.2
Culicoides 71189/1	68638.03	4.8	97.20	4.6	0.2
Culicoides 71189/6	44221.17	4.6	48.15	3.9	0.7
Culicoides 71189/7	44530.87	4.6	22.95	3.6	1.0
Culicoides 71820/6	257642.51	5.4	96.08	4.8	0.6
Culicoides 71820/8	1090.00	3.0	5.18	1.9	1.1
Culicoides 72678/1	3625.14	3.6	8140.05	3.9	−0.3
Culicoides 72678/4	119916.18	5.1	72.90	4.5	0.6
Culicoides 72678/5	3642.75	3.6	4250.00	3.6	0.0
Culicoides 72678/7	144674.11	5.2	48.83	4.5	0.7
Blood 79384/1	1625.47	3.2	1433.25	3.2	0.0
Blood 79384/2	36.93	1.6	26.30	1.4	0.2
Blood 79384/3	160.06	2.2	117.90	2.1	0.1
Blood 79386/1	1187.62	3.1	695.25	2.8	0.0
Blood 79386/2	546.29	2.7	155.70	2.2	0.5
Blood 79386/3	1291.15	3.1	409.50	2.6	0.5
Blood 79390/1	168.73	2.2	101.70	2.0	0.2
Blood 79390/2	2712.23	3.4	191.25	2.3	1.1
Blood 79390/3	935.12	3.0	621.00	2.8	0.2
Blood 79364/1	36.02	1.6	18.00	1.3	0.3
Blood 79364/2	136.48	2.1	30.15	1.5	0.6
Blood 79364/3	100.49	2.0	57.82	1.8	0.2
Blood 127	2571.00	3.4	2944.35	3.5	−0.1
Blood 236	9.00	1.0	2.70	0.4	0.5
Blood 341	88.00	1.9	73.35	1.9	0.1
Blood 449	1550.00	3.2	806.85	2.9	0.3
Blood 695	390.00	2.6	226.35	2.4	0.2
Blood 8123	5.00	0.7	4.50	0.7	0.0
Blood 9124	1599.00	3.2	1134.00	3.1	0.1
Blood 11173	106.00	2.0	108.45	2.0	0.0
Blood 13247	577.00	2.8	264.15	2.4	0.3
Blood 14262	60.00	1.8	24.75	1.4	0.4
Blood 15278	11.00	1.0	11.70	1.1	0.0
Blood 16279	24750.00	4.4	8892.00	3.9	0.4
Blood 17290	20904.00	4.3	12326.85	4.1	0.2
Blood 18296	63.00	1.8	44.55	1.6	0.2
Blood 19301	66.00	1.8	252.00	2.4	−0.6
Blood 20307	1.00	0.0	0.45	−0.3	0.3

a *Field samples analyzed in RT-qPCRNS_3_ and RT-ddPCR*.

b *copies BTV/μl of sample in RT-qPCRNS_3_*.

c *Log of copies number BTV in μl/sample in RT-qPCRNS_3_ assay*.

d *copies BTV/μl of sample in RT-ddPCR*.

e *Log of copies number BTV in μl/sample in RT-ddPCR assay*.

f *Log (RT- qPCRNS_3_ quantification) – Log (RT-ddPCR quantification)*.

## Discussion

BTV is responsible for an important disease of ruminant that induces variable clinical signs, its pathogenicity depends on the host species. Seasonal incursions of the disease in parts of Europe (Mediterranean basin) during the summer cause economic losses due to direct impact on livestock and trade restriction. In the last few years, new serotypes have been identified, probably, originated under evolutionary dynamics and selection pressure (37); new potential vectors have been identified (38, 39); some field strains/serotypes proved to be able to transmit vertically or horizontally, to reassort their RNA, and to alter their pathogenicity, specificity, and spread capacity (37). Nowadays, the molecular diagnosis of BTV (real time RT-PCR_NS3_) by Hofmann allows to identify all circulating serotypes. These knowledges designate a complex contest in which it is necessary to have more sensitive and accurate methods, not only to assess the presence/absence of the virus but also to evaluate the RNA viral load in natural and/or experimental infected samples. In this study, we established a novel RT-ddPCR assay for the quantification of RNA BTV at low concentration of virus and in spiked and field samples including whole blood, tissues, and midges, showing the power and the potential of RT-ddPCR assay. This assay has been enabled to detect absolute target copy number in field samples without employing recombinant DNA or RNA plasmid to construct the standard curve. As a consequence, the amplification efficiency bias observed with quantitative RT-qPCR is minimized. A certified reference material (CRM) for BTV quantification is not available; therefore, further independent quantification methods are needed in order to quantify the BT virus as number of copies/μl sample, in the greatest way possible. NS3 gene primers and probe sequences used in RT-ddPCR assay were from the previously published BT reverse transcriptase real-time PCR (RT-PCR_NS3_) by Hofmann et al. (21) and OIE (34), mentioned above for diagnostic purposes. RT-qPCR_NS3_ is performed using degenerate primer and probe set that detect all serotypes of BTV; RT-ddPCRs were done with some specific modifications in order to optimize all parameters. RT-ddPCR assay exhibited a good degree of linearity (*R*^2^ ≥ 0.995) in the range from 10^2.43^TCID_50_/ml to 10^−0.57^ TCID_50_/ml, overcoming the dependence on the availability of references or standards. BTV-1 10^2.43^TCID_50_/ml was considered the first point of dynamic range. Evaluating the dynamic range of the digital assay, the 10^3.43^ TCID_50_/ml viral dilution (corresponding to Cq 22.5 in RT-qPCR_NS3_) was excessively concentrated to be detectable because all of the droplets were positive (saturation of the reaction). In contrast, the last point of the range (BTV-1 10^−1.57^ TCID_50_/ml) was excessively diluted (data not shown); then the last point was 10^−0.57^ TCID_50_/ml viral dilution. As expected, the dynamic ranges of newly RT-ddPCR were lower (four orders of magnitude) than those obtained for the corresponding qPCRs for all samples. Several studies reported that digital PCR shows higher tolerance to inhibitors; as an end-point measurement, it can reduce the biases linked to matrix type often observed with qPCR (40), especially in clinical specimen (stool, sputum, tissue) (16, 17, 41). Pavšič et al. (42) suggested that it may be possible to perform ddPCR on samples without extraction of nucleic acid. Quantitative RT-ddPCR data obtained from blood and spleen showed a good level of tolerance to inhibitors when compared to the same TCID_50_/ml BTV-1 dilutions with a good linearity close to 1. Despite pGEM plasmid not being a properly standard reference, the use of serial dilutions of the plasmid allowed us to evaluate the precision of the RT-ddPCR for quantification purposes. These results suggest that RT-ddPCR assay provides an accurate quantification of BTV, unevenly distributed in different matrices, without precluding the quantitation efficiency due to impurities in the sample. The LoD and the LoQ were established at 10^−0.67^ TCID_50_/ml and 10^0.03^ TCID_50_/ml of BTV-1 corresponding to 0.72 and 3.05 copies/μl, respectively. Data of intra- and inter-assay repeatability of the RT-ddPCR showed a good repeatability with a variability below the threshold (CV 25%) for acceptance criteria of quantitative methods (35, 36) and were assessed in two different days with <13% variability between the results. The good precision of RT-ddPCR is linked to the intrinsic characteristics of the method, enabling absolute quantification of the viral target at different work conditions. In the last part of this study, we compared the RT-ddPCR against RT-qPCR_NS3_ using 44 field samples that resulted positive to real-time RT-PCR_NS3_. Applicability of the technology has been tested on characteristic matrices of field and on the range of viral load generally distributed in routine samples. The collected whole blood samples, tested as positive with the official method, stored at 4°C, and selected for comparative study with both assays, showed the same Cq found in RT-qPCR_NS3_ during the diagnosis (data not shown). These results support a previous work in which the persistence of BTV in stored blood samples were observed (43). The main limitation of RT-ddPCR respect to RT-qPCR_NS3_ was that the higher concentration of template resulted in saturation of positive droplets, confirming the saturation at high concentration of RNA. Thus, the concentrated samples were diluted for viral load quantification in RT-ddPCR. The results showed a log difference average of 0.30 in field samples. A higher Seg-10 BTV-1 quantification by RT-qPCR_NS3_ could be addressed to the C_q_ values that are established on transcription and amplification efficiency. This could affect the RT-qPCR, but does not have any effects on digital PCR. Anyway, the analysis of correlation demonstrated that both assays provided similar measurements with a very close agreement between the systems. On the other hand, the main advantage of the RT-ddPCR_NS3_ is the ability to quantify any BTV serotype without the use of standard curves properly constructed for each specific group. Moreover, the implementation of the serotype-specific multiplexing system should be suitable to detect and quantify simultaneously different BTV serotypes in case of viral coinfection in areas that are circulating different BTV serotypes. Despite its general advantages, compared to qPCR, dPCR is more time consuming and labor intensive, but will certainly give further, in terms of applicability and throughput.

## Data Availability Statement

The datasets generated for this study are available on request to the corresponding author.

## Ethics Statement

The study did not involve any animal experiment. Samples were collected from sheep with standard procedures by the Sardinian Veterinary Services A.T.S. and submitted to the Experimental Zooprophylactic Institute of Sardinia for BTV testing. Special authorization for sampling activities was not necessary; these actions are regulated by the Italian Ministry of Health and performed in case of BT outbreaks.

## Author Contributions

MT, AR, and RB carried out experimental work. AR and MT wrote the first draft of the manuscript with support from GP and MD. All authors contributed to conception and design of the study, data analysis and interpretation, manuscript revision, and read and approved the submitted version.

## Conflict of Interest

The authors declare that the research was conducted in the absence of any commercial or financial relationships that could be construed as a potential conflict of interest.
